# Diffusion-Weighted Magnetic Resonance Imaging (dMRI) and Cochlear Implant Outcomes in Axonal Auditory Neuropathy: A Case Report

**DOI:** 10.3390/jcm13113072

**Published:** 2024-05-24

**Authors:** Gary Rance, Raoul Wills, Andrew Kornberg, Julien Zanin

**Affiliations:** 1 Department of Audiology and Speech Pathology, The University of Melbourne, Carlton, VIC 3052, Australia; julien.zanin@unimelb.edu.au; 2 Cochlear Implant Clinic, The Royal Victorian Eye and Ear Hospital, East Melbourne, VIC 3002, Australia; raoul.wills@eyeandear.org.au; 3 Department of Neurology, Royal Children’s Hospital, Parkville, VIC 3052, Australia; andrew.kornberg@rch.org.au; 4 Murdoch Children’s Research Institute, Parkville, VIC 3052, Australia; 5 Department of Paediatrics, Faculty of Medicine, Dentistry and Health Sciences, University of Melbourne, Parkville, VIC 3052, Australia

**Keywords:** diffusion-weighted magnetic resonance imaging, riboflavin transporter deficiency, auditory neuropathy, axonopathy

## Abstract

Background: Progressive auditory dysfunction is common in patients with generalized neurodegenerative conditions, but clinicians currently lack the diagnostic tools to determine the location/degree of the pathology and, hence, to provide appropriate intervention. In this study, we present the white-matter microstructure measurements derived from a novel diffusion-weighted magnetic resonance imaging (dMRI) technique in a patient with axonal auditory neuropathy and consider the findings in relation to the auditory intervention outcomes. Methods: We tracked the hearing changes in an adolescent with Riboflavin Transporter Deficiency (Type 2), evaluating the sound detection/discrimination, auditory evoked potentials, and both structural- and diffusion-weighted MRI findings over a 3-year period. In addition, we explored the effect of bilateral cochlear implantation in this individual. Results: Between the ages of 15 years and 18 years, the patient showed a complete loss of functional hearing ability. The auditory brainstem response testing indicated an auditory neuropathy with evidence of normal cochlear function but disrupted auditory neural activity. While three structural MRI assessments across this period showed a clinically normal cochleovestibular anatomy, the dMRI evaluation revealed a significant loss of fiber density consistent with axonopathy. The subsequent cochlear implant function was affected with the high levels of current required to elicit auditory sensations and concomitant vestibular and facial nerve stimulation issues. Conclusions: The case study demonstrates the ability of dMRI technologies to identify the subtle white-matter microstructure changes in the auditory pathway, which may disrupt the neural function in patients with auditory axonopathy.

## 1. Introduction

Auditory neuropathy (AN) is a hearing abnormality involving normal peripheral (cochlear outer hair cell) function but disordered neural activity in the VIII^th^ cranial nerve and central auditory brainstem [1]. The disorder is associated with a number of etiologies affecting different sites in the auditory pathway, but there are two primary pathological mechanisms: deafferentiation and dyssynchrony. Deafferentiation involves a reduction in the number of activated nerve fibers and is common in diseases associated with auditory nerve axonopathy, such as Friedreich ataxia, Charcot–Marie–Tooth disease (Type 2), and Riboflavin Transporter Deficiency. Dyssynchrony, in contrast, occurs when the consistency (timing) of the neural firing is affected and is most commonly observed in demyelinating conditions such as Charcot–Marie–Tooth disease (Type 1) [1].

Auditory neuropathy may present in infancy, where it is associated with neonatal insults such as hypoxia and hyperbilirubinemia [1]. As such, it is relatively common in NICU graduates and accounts for approximately 10% of the cases of permanent childhood hearing impairment. There are also progressive forms that typically present in adolescence/early adulthood and are often associated with generalized neurodegenerative conditions [1].

Individuals with both the perinatal and progressive forms suffer severe functional hearing difficulties due to the disruption of the auditory neural code. This results in temporal distortion, which can render complex acoustic signals (such as speech) unintelligible—even when the sounds are clearly audible [2]. This neural distortion makes intervention problematic as conventional hearing aids make sounds louder but not clearer for affected individuals. Cochlear implantation is often successful, but the outcomes are dependent on the site of the lesion and degree of neural disruption [1]. In particular, a pathology occurring at or beyond the cochlear nucleus may limit the perception as the CI-generated signal must still be transmitted through a disordered auditory neural system.

While the recent advances have enabled the accurate localization of the site of the lesion in some genetic forms of auditory neuropathy, clinicians currently lack the diagnostic tools to determine the location and degree of the auditory pathway abnormality and, hence, to predict the intervention outcomes in most cases.

In this case report, we present the findings for an adolescent girl (Patient 1) admitted to the Neuroaudiology Clinic at the University of Melbourne with an 18-month history of hearing difficulty. She had shown no risk factors for pediatric hearing impairment and followed a standard developmental course, meeting the speech, language, and academic milestones through the pre-adolescent period.

## 2. Detailed Case Description

**Figure 1:**
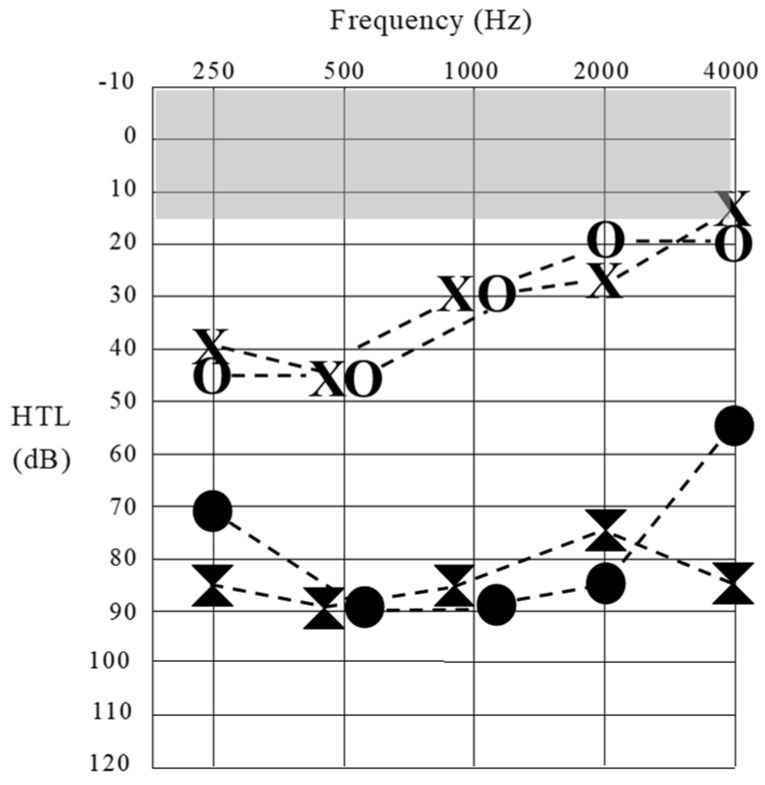
Sound detection thresholds obtained for Patient 1 at octave frequencies across the audiometric range. Results at 16 years of age are represented by unfilled data points (left ear [X] and right ear [O]). Threshold levels at 18 years of age are represented by filled data points. The shaded (gray) area shows the normal sound detection threshold range.

At age 15 years, Patient 1 began experiencing hearing/communication problems in background noise and reported an inability to localize sound sources. An audiometric assessment subsequently revealed mildly impaired sound detection—particularly for low-frequency stimuli (Figure 1). The functional hearing ability (speech perception) was, however, negligible. On open-set word testing, she was able to identify 40% of the phonemes presented auditorily/visually but scored 0% for stimuli presented auditorily alone. A tympanometric assessment revealed normal middle ear function, and structural magnetic response imaging (MRI) indicated normal cochlear structure and auditory nerve anatomy bilaterally. At no stage did Patient 1 experience balance disturbance, and, as such, she did not undergo vestibular assessment. She was subsequently fitted with conventional hearing aids, which afforded her complete access to the speech sounds at normal voice levels—but no perceptual benefit. By age 16 years, she had abandoned the amplification, had become non-verbal at school and in most social situations, and was communicating primarily through signs/gestures and telephone texting.

## 3. Neuroaudiology

**Figure 2:**
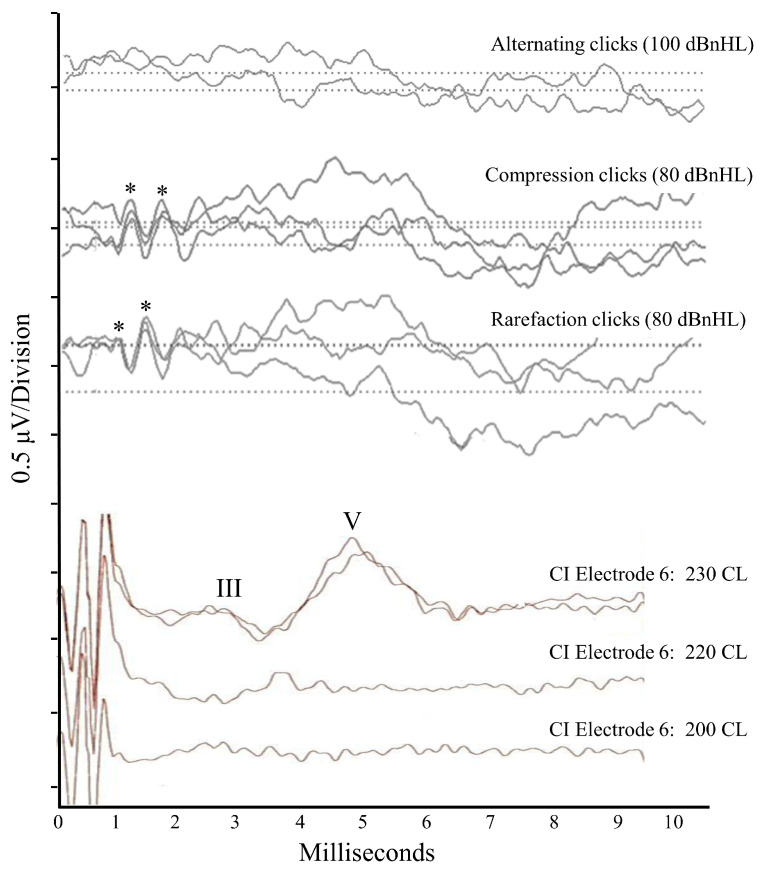
Scalp-recorded electroencephalogic responses for Patient 1. The top three tracings were obtained for acoustic stimuli presented to the right ear when the patient was 16 years of age. The first tracing shows no response to alternating polarity click stimuli at 100 dBnHL. The second and third tracings show absent ABRs but present cochlear microphonic responses to unipolar (compression or rarefaction) clicks at 80 dBnHL. Asterisks denote the positive peaks in the microphonic waveform. The bottom three tracings were obtained intraoperatively (18 years of age) to electrical pulses generated on Electrode #6 of a multichannel device implanted into the right cochlea. Stimulation at the maximum level (230 current levels) shows a repeatable auditory brainstem response (positive peaks for Wave III and Wave V marked), while presentation at lower levels was sub-threshold.

The neuroradiological assessment at the age of 16 years revealed the classic AN result pattern, with evidence of pre-neural auditory activity and absent neural responses. Normal cochlear-level physiology was indicated in both ears by the presence of Distortion Product Otoacoustic Emissions (which reflect the mechanical function of the outer hair cells) and cochlear microphonic responses (which are generated by the polarization/depolarization of hair cells with the movement of the basilar membrane) (Figure 2). Bilateral auditory neural dysfunction in the VIII^th^ nerve and brainstem was indicated by the absence of scalp-recorded auditory evoked potentials (auditory brainstem response [ABR]) to acoustic stimuli at the maximum presentation levels in each ear (Figure 2).

The functional hearing ability was severely impaired. The perception of sentences in background noise (LiSN-S test) was negligible in listening conditions consistent with everyday communication environments (school classrooms, shopping centers, etc.). Furthermore, she showed no binaural processing ability—i.e., the capacity to combine the inputs from the two ears to localize sound sources and improve perception in background noise. These findings were significantly poorer than expected for her (mild) degree of hearing loss and are consistent with those observed in AN patients with distorted neural representations of acoustic timing cues [3].

## 4. Diagnosis

Following the identification of the auditory neuropathy, Patient 1 was referred for a neurological opinion. The examination at age 16 years was unremarkable, as were the further investigations, including nerve conduction studies (NCSs) and electromyography (EMG). The standard MRI of the brain was considered to be normal. She had progression in her symptoms over the subsequent 4 years, with weight loss and the development of a foot drop. She developed weakness in her hands with subsequent associated limb weakness and breathlessness. A repeated EMG at age 20 years showed signs of a mixed upper and lower motor neuron abnormality.

Interestingly, Patient 1’s auditory dysfunction became apparent 2–3 years before the presentation of motor and other symptoms. This is not uncommon in generalized neurodegenerative conditions and has been reported previously for other diseases, such as Friedreich ataxia and Charcot–Marie–Tooth disease [1]. The auditory pathway is uniquely sensitive to neural disruption as the perception of speech and localization of sound direction are dependent on the precise representation of rapidly changing acoustic signals [2]. As such, it has been suggested that auditory measures may be useful as biomarkers for neurodegenerative conditions, capable of tracking the natural history of disease [1] and identifying subtle changes occurring as a result of pharmacological intervention [4].

The genetic assessment at age 19 years identified a pathologic variant, c.751C > T (p.Gln251*), in the gene SLC52A2, which is associated with autosomal recessive Riboflavin Transporter Deficiency neuronopathy (RTD2)—also known as Brown–Vialetto–Van Laere syndrome 2 [5]. RTD2 is a rare autosomal recessive neurological disorder characterized by motor, sensory, and cranial nerve neuropathy [5,6]. While the onset occurs most frequently in childhood, occasionally, the condition presents in early-to-mid adulthood [5]. Auditory perceptual difficulties, including elevated hearing thresholds, are often the first symptoms; however, as the disease progresses, the affected individuals develop muscle weakness (including respiratory insufficiency), sensory ataxia, and vision loss [5,6]. The prevalence rates remain unclear due to underdiagnosis and variable clinical presentation; however, it is estimated that RTD2 affects less than 1 per million [7]. The disease is primarily caused by biallelic loss-of-function mutations in the genes involved in riboflavin metabolism, particularly the *SLC52A2* and *SLC52A3* genes, which encode riboflavin transporter proteins and play a crucial role in cellular metabolism and neuronal integrity [5]. Consequently, the impaired transport of riboflavin across cell membranes has been shown to cause mitochondrial dysfunction and oxidative stress, leading to neuronal cell death and directly contributing to the neurological manifestations observed in RTD2 [5,6].

## 5. Imaging

**Figure 3:**
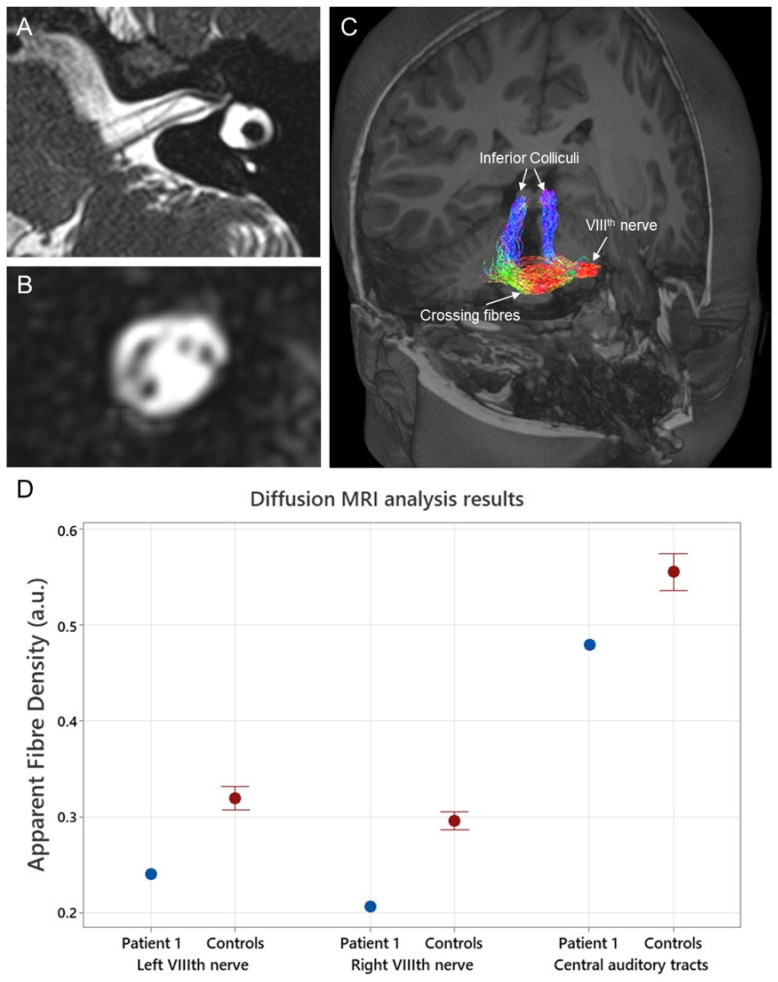
(**A**) High-resolution T2-weighted (3D SPACE) structural MR image (sequence parameters include voxel size = 0.3 × 0.3 × 0.3 mm^3^, repetition/echo time = 1000/141 ms, and flip angle = 120°) showing an axial slice of Patient 1’s left internal auditory canal. (**B**) A coronal slice through the internal auditory canal of the same image shown in (**A**). The cochlear, facial, superior, and inferior vestibular nerves are visible. (**C**) 3-dimensional render of a T1-weighted image with an overlay of Patient 1’s auditory tracts from the left VIII^th^ nerve to the inferior colliculus bilaterally. These tracts were generated using the information obtained from diffusion-weighted MRI (sequence parameters 2.5 mm isotropic voxels, repetition/echo time = 8400/110 ms, matrix size = 96 × 96, and acceleration factor of 2) with probabilistic tractography. Quantitative metrics were then generated from two segments of these tracts (VIII^th^ nerve and ascending tracts). The auditory tracts are color-coded according to direction: left–right is red, anterior–posterior is green, and superior–inferior is blue. (**D**) Apparent fiber density results, a quantitative measure of axonal fiber density, obtained from Patient 1 and a group of neurologically normal controls (*n* = 21). Central auditory tract data were averaged across both ears. The MRtrix3tissue software package version v5.2.9 (https://3tissue.github.io/ accessed on 10 February 2024) was used to analyze the diffusion data. Apparent fiber density values are expressed in arbitrary units (a.u.).

Patient 1 underwent three structural MRI assessments (aged 16 years, 17 years, and 18 years) through her period of auditory deterioration. Each of these was qualitatively assessed and considered (by experienced radiologists/ENT surgeons) to show unremarkable cochlear anatomy and structurally normal cochleovestibular nerves with normal (or perhaps slightly reduced) neural volumes. Figure 3A,B show the results from the structural MRI obtained from Patient 1 at 18 years as part of the pre-operative cochlear implant candidacy assessment.

Diffusion-weighted MRI (dMRI) was undertaken at age 17 years. Diffusion-weighted MRI is currently the only non-invasive method available to study white matter (WM) microstructures and connectivity in vivo [8]. It is a data acquisition strategy, available on clinical MRI scanners, that can measure the diffusion orientation of the water molecules within the brain. Since the pattern of diffusion is markedly different in WM compared to grey matter or cerebrospinal fluid, mathematical models can use this information to provide insights into the orientation and organization of the WM fiber pathways [8]. In addition, the quantitative metrics of the WM microstructure can be derived, which provide an indication of the density of the axon fibers within a specific WM bundle. The quantitative metrics of the fiber density offer an objective alternative to the subjective visual examination of the VIII^th^ nerve relied upon in structural MRI analysis. This could be advantageous as the subjective interpretation of imaging results may vary depending on clinician experience [9]. Diffusion-weighted MRI has previously been successful in identifying the axonal neuropathy affecting the VIII^th^ nerve in individuals with X-linked auditory neuropathy [10], where the VIII^th^ nerve fiber density results obtained from affected individuals have been correlated with perceptual ability.

Diffusion-weighted MRI data were acquired using a 3-Tesla Siemens Magnetom Skyra system, a 32-channel head coil receiver, and the following echo planar imaging sequence parameters: 2.5 mm isotropic voxels, repetition/echo time = 8400/110 ms, matrix size = 96 × 96, and acceleration factor of 2. In total, 64 diffusion-weighted images using a high diffusion weighting (b = 3000 s/mm^2^) and 8 non-diffusion (b = 0 s/mm^2^) images were acquired. The dMRI results for Patient 1 were analyzed using the MRtrix3tissue v5.2.9 software package (https://3tissue.github.io/ accessed on 10 February 2024). Apparent fiber density values, a quantitative measure of axonal fiber density, were extracted from Patient 1’s VIII^th^ nerve and their central ascending auditory tracts (cochlear nucleus to inferior colliculus) (Figure 3C). While fiber density values were extracted separately from the left and right VIII^th^ nerves, the central auditory tracts were averaged across both ears due to the substantial amount of crossing fibers [10]. Results were compared to fiber density results from 21 neurologically normal controls. Eighth nerve fiber populations from Patient 1 were markedly reduced compared to controls (Figure 3D). The apparent fiber density for the left VIII^th^ nerve in Patient 1 was 0.24 (z-score −3.05), and, for the right, it was 0.21 (z-score −4.35). In contrast, the apparent fiber density values of Patient 1’s central ascending tracts were relatively unaffected (apparent fiber density 0.49; z-score −1.8). These findings are consistent with post-mortem histological results in RTD2, which have shown specific neuronal loss in the VIII^th^ cranial nerve [5].

## 6. Cochlear Implantation

Between the ages of 16 years and 18 years, Patient 1 showed a progressive loss of sound detection ability (Figure 1) and negligible functional hearing. As such, she was referred to the Melbourne Cochlear Implant Clinic, and, despite the fact that individuals with RTD2 have shown variable CI outcomes [11]), she was fitted with multichannel cochlear implant devices at age 18 years (right ear) and 22 years (left ear).

For the right ear, she was implanted with a straight electrode array (Cochlear CI522). The surgery was uneventful, and all the stimulating electrodes were sited within the cochlear partition. The intraoperatively evoked potential test results (using CI-generated stimuli to elicit auditory pathway responses) were, however, abnormal. The electrically evoked compound action potentials (ECAPs) were unrecordable. These near-field responses, recorded using adjacent electrodes in the CI array, reflect the first action potential in the auditory nerve and were absent to electrical pulses at the maximum levels for each of the stimulating electrodes. This result is unusual, with <4% of the patients with normal cochlear anatomy showing absent potentials [12], and is consistent with a paucity of auditory nerve fibers.

Similarly, the electrically evoked Auditory Brainstem Responses (EABRs) were atypical. While ABR waveforms of normal latency and morphology could be elicited (Figure 2), responses were only observed to stimuli at abnormally high stimulus current levels [13]. The fact that a repeatable waveform could be elicited at all in a patient with auditory neuropathy is significant and indicates that synchronized neural firing could be evoked in the late brainstem once sufficient levels of current were provided. As such, the presence of the EABRs is indicative of axonopathy as the mechanism causing the patient’s auditory neuropathy. Desynchronizing pathologies, in contrast, affect the consistency of neural activity and prevent ABR recording regardless of the stimulus presentation level [2]. The presence of the CI-evoked ABRs is also consistent with the dMRI finding that the auditory neural elements between the cochlear nucleus and lateral lemniscus were unaffected in Patient 1 by the RTD2 disease process (Figure 3).

The post-operative device programming also required extreme levels of current to elicit auditory sensations and/or to achieve growth in loudness. This increased current requirement creates a range of technical challenges, including a significant reduction in device battery life. More importantly, high stimulation levels result in less localized current flow and increased risk of non-auditory sensation. Patient 1 experienced both eye twitching (indicating facial nerve stimulation) and dizziness (suggesting vestibular stimulation) with device use—both of which occur in <1% of the patients with CI stimulation at the typical levels [14]. Neither of these symptoms had been observed pre-operatively or post-operatively when the device was not in use. These manifestations were alleviated by increasing the implant signal pulse width (i.e., extending the duration of each stimulus to reduce the peak level of current required to elicit an auditory sensation). In this case, the pulse width had to be increased from the standard 25–37 µs to 100 µs to provide an audible and comfortable stimulus program. The manipulation of the CI stimulus parameters in this way does, however, have potential limitations as the wider pulse width slows the overall stimulation rate, which in turn limits the amount of information that can be presented and (potentially) affects perception [15]. For Patient 1, the stimulation rate needed to be reduced from the (Cochlear Ltd., Macquarie Park, Australia) default recommendation of 900 Hz to only 500 Hz. This allowed all the other device parameters (the number of stimulus maxima, etc.) to be maintained at the default settings.

Given these device programming issues, Patient 1 was provided with a curved electrode array (Cochlear CI632) when implanted on the left side. The aim here was to have the electrodes hug the cochlear modiolus and, hence, to reduce the amount of current required (and potential current spread) through their proximity to the neural elements contained therein. This strategy was unsuccessful. Electrical compound action potentials were again absent at the maximum stimulus levels, and the behavioral device programming required stimuli with 100 µs pulse widths to produce auditory sensations.

Despite the programming challenges, the functional hearing ability for Patient 1 was relatively normal. She showed binaural speech perception scores (i.e., speech reception thresholds for sentences in background noise) around 7–8 dB at 12 months following the second implant, which is within the expected CI performance range [16]. Furthermore, at the time of writing, she was a consistent device user, employing both implants in a range of listening and communication contexts. This outcome (which is not reflective of all CI recipients with auditory neuropathy) does fit with our evoked potential and dMRI findings, which have indicated that synchronized neural firing could be elicited by the implant and that the fiber density in the central auditory pathways had not been affected by the RTD disease.

## 7. Study Limitations

As always with single-patient investigations, the study conclusions need to be interpreted with care. While the findings for Patient 1 were compared with published normative data sets, we did not provide a precisely matched control. Future studies should involve larger patient groups and cohorts of healthy gender- and age-matched controls with varying degrees and types of hearing loss. Furthermore, the pre-operative dMRI findings in CI recipients with a range of outcomes should be compared to establish the predictive capacity of this objective imaging technique.

## 8. Conclusions

In summary, the findings of this case demonstrate the potential clinical applications afforded by dMRI technologies. Where the current gold standard (structural MRI) suggested clinically normal vestibulocochlear nerves throughout the period of hearing degeneration (15–18 years), dMRI revealed the presence of reduced fiber density (specific to the vestibulocochlear nerve), which was consistent with the RTD2 etiology. This enervation deficit had significant effects on the cochlear implant function (i.e., high current levels and concomitant vestibular and facial nerve stimulation issues), which might have been anticipated from her fiber density levels. While further studies are required to elucidate the relationship between the dMRI measures of auditory neural anatomy and cochlear implant (perceptual) outcomes, this case offers a unique example of imaging technologies predicting the electrical stimulability of the VIII^th^ nerve and central auditory pathways. Hence, incorporating dMRI alongside the current high-resolution T2-weighted structural imaging protocol could enhance the assessment of cochlear implant candidacy. Both scans could feasibly be acquired in a single session with minimal additional scanning time. In this way, structural MRI could be used to assess the cochlear anatomy and guide the surgical planning, whereas dMRI would aid in determining the candidacy, particularly in identifying a viable nerve, especially in cases where the subjective visualization of the cochlear partition is inconclusive. It may also prove useful in determining which ear should be implanted in situations where bilateral implantation is not standard.

## Data Availability

All data underlying the results are available as part of the article, and no additional source materials are required.
